# Supplementary material for: Expanding the Toolbox for Functional Genomics in Fonsecaea pedrosoi: The Use of Split-Marker and Biolistic Transformation for Inactivation of Tryptophan Synthase (trpB) Gene
Source: J Fungi (Basel). 2023 Feb 8;9(2):224. doi: 10.3390/jof9020224 (PMC9963410; doi:10.3390/jof9020224)
Supplement: Supplementary file 1 [file jof-09-00224-s001.zip › jof-2172503-supplementary.pdf]

Favilla, L.D. Supplementary Materials

Table S1. Oligonucleotides used in this work

Name	Target	Use	Sequence 5´ -3´
**Fp15**	Hyg^R	DJ-PCR/ Deletion confirmation	CGTCTGCTGCTCCATACAAGC
**Fp16**	Hyg^R	DJ-PCR/ Deletion confirmation	CATATGCGCGATTGCTGATCC
**Fp90**	Hyg^R	DJ-PCR	CCGCGACGTTAACTGATATT
**Fp91**	Hyg^R	DJ-PCR	GTGGAGCCAAGAGCGGATT
**Fp118**	3´ *trpB*	Deletion confirmation	GTCCCGGTGTCTACTATGAA
**Fp119**	5´ *trpB*	Deletion confirmation	ATCGGTGTACAAGGCAAGC
**Fp120**	ORF *trpB*	Deletion confirmation	AGGGAGTGGTGATTGGTAGT
**Fp121**	ORF *trpB*	Deletion confirmation	CAGTGTGATTCAGGTCCTCT
**Fp128**	5´ *trpB*	DJ-PCR	GGGCATATGTTCTTCCAGGAC
**Fp129**	5´ *trpB*	DJ-PCR	AATATCAGTTAACGTCGCGGGTCAAGGTTGAAAGGGA
**Fp130**	3´ *trpB*	DJ-PCR	AATCCGCTCTTGGCTCCACACGGCGTCAACCTCAAGATA
**Fp131**	3´ *trpB*	DJ-PCR	CACAGCTCGACGGATAGGATA
**Fp217**	*trpB*	RT-PCR	CTCATCCCTTCCCGACGAT
**Fp218**	*trpB*	RT-PCR	CCACCGACACAAGCAATCAC
**Fp219**	*trpC*	RT-PCR	TGCCCTTCTCGTCGGTGTAT
**Fp220**	*trpC*	RT-PCR	CCGCATCCAATCCAAACTTC
**Fp221**	*trpD*	RT-PCR	GAAGTCGTGCGGAAGAGGAA
**Fp222**	*trpD*	RT-PCR	CGTGGTCGAGATGTTGAATGTC
**Fp223**	*trpE*	RT-PCR	TTCCTCTGCCCTACCAACCA
**Fp224**	*trpE*	RT-PCR	ACGTGCGCCTCATATCCTGT
**Fp225**	*gapdh*	RT-PCR	AAGGGTGGTGCGAAGAAGGT
**Fp226**	*gapdh*	RT-PCR	GGGATGTCGGGCTTGTATGA

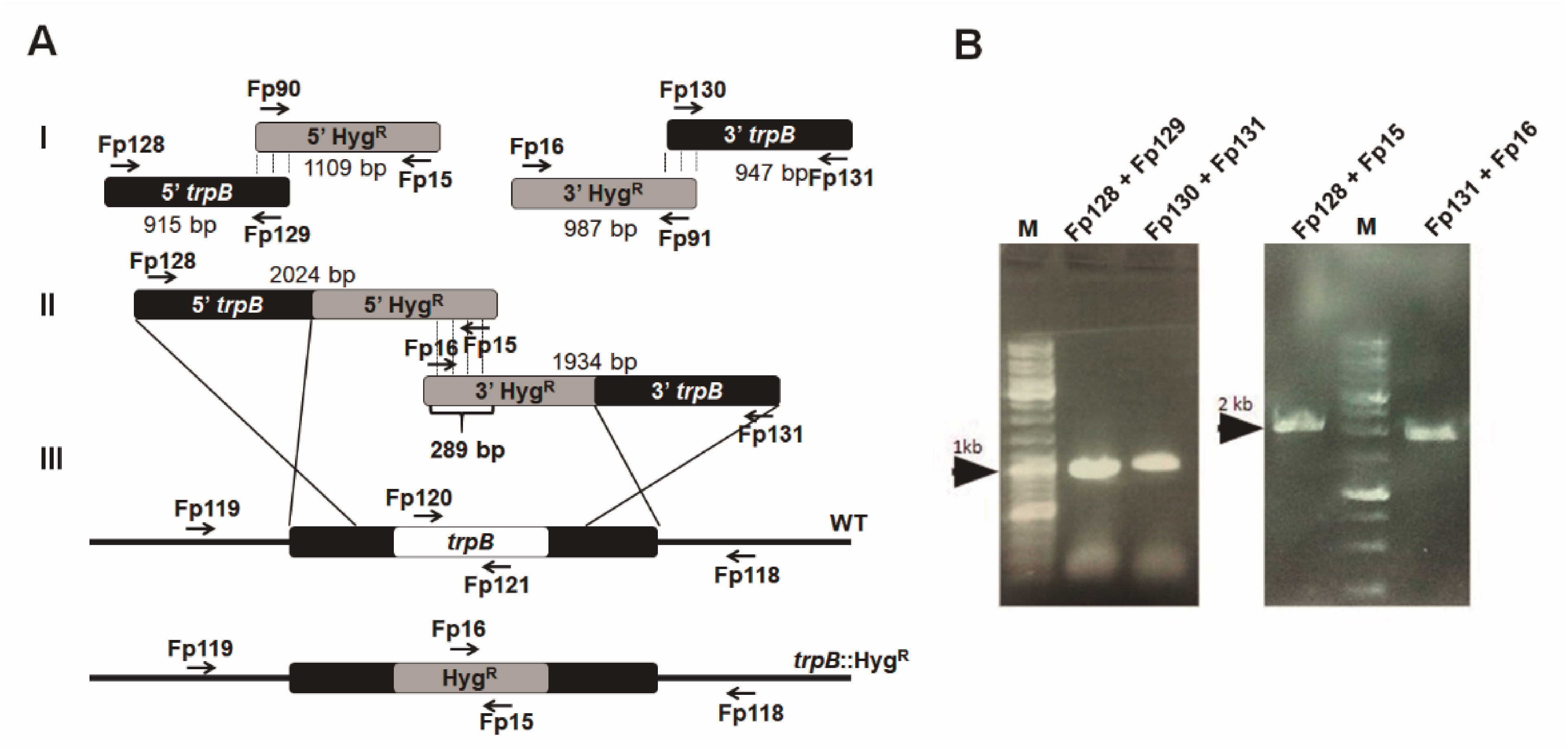

Figure S1. Construction of *trpB* disruption cassette by DJ-PCR/Hyg^R split marker strategy. (A) The first step of PCR generated four fragments, corresponding to the 5' and 3' flanking regions of the *trpB* and of Hyg^R selective marker. *trpB* 5' and 3' were amplified on reactions Fp128+Fp129, and Fp130+Fp131, respectively. Hyg^R 5'and 3' were obtained by Fp90+Fp15 and Fp16+Fp91, respectively (amplicons not shown in the figure B). The second reaction generated two overlapping fragments: 5' *trpB*/Hyg^R (Fp128+Fp15) and 3' *trpB*/Hyg^R (Fp16+Fp131) that were directly transformed on *F.pedrosoi* spores. (B) The DJ-PCR products for deletion cassette construction were electrophoresed in TAE 1× agarose gel in order to check the expected sizes. The length of the amplicons are depicted on (A). M: Molecular weight marker.

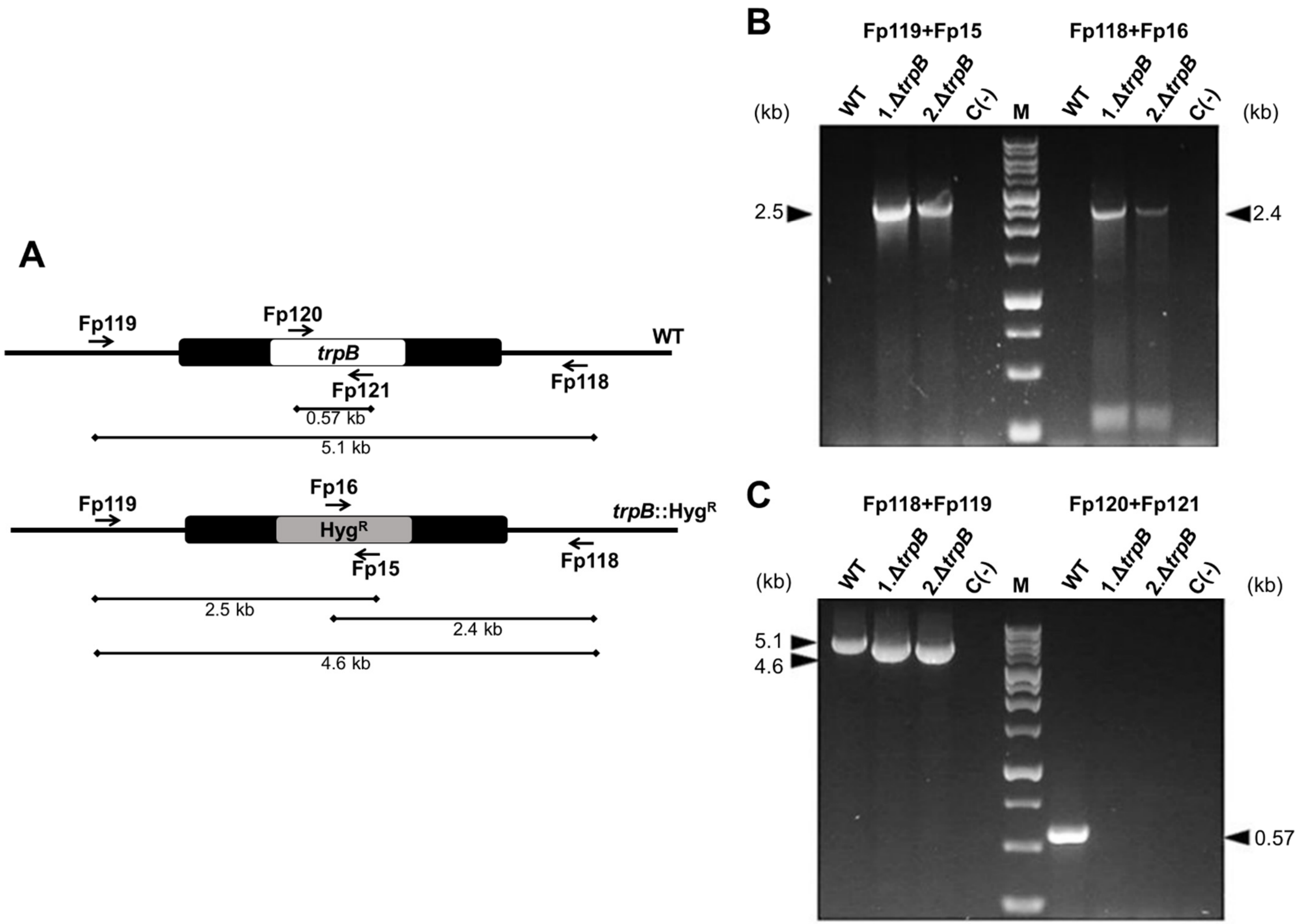

Figure S2. PCR checking for homologous recombination of the *trpB* disruption cassette. (A) Scheme representing the oligonucleotide positions used to confirm the insertion of Hyg^R into *trpB* locus of *F. pedrosoi*. (B) Products obtained after 5’ and 3’ *trpB* locus amplification confirmed Hyg^R cassette insertion. (C) On the left are shown the amplification of the full locus generating a 4.6kb for *trpB::hph* replacement and a 5.1kb related to intact allele. On the right side, internal oligonucleotides specific to *trpB* evidenced a 570 bp product in WT strain indicating the presence of *trpB* allele. The expected product sizes are depicted in the picture. M: Molecular weight marker, WT: wild type strain, C (-): H_2O

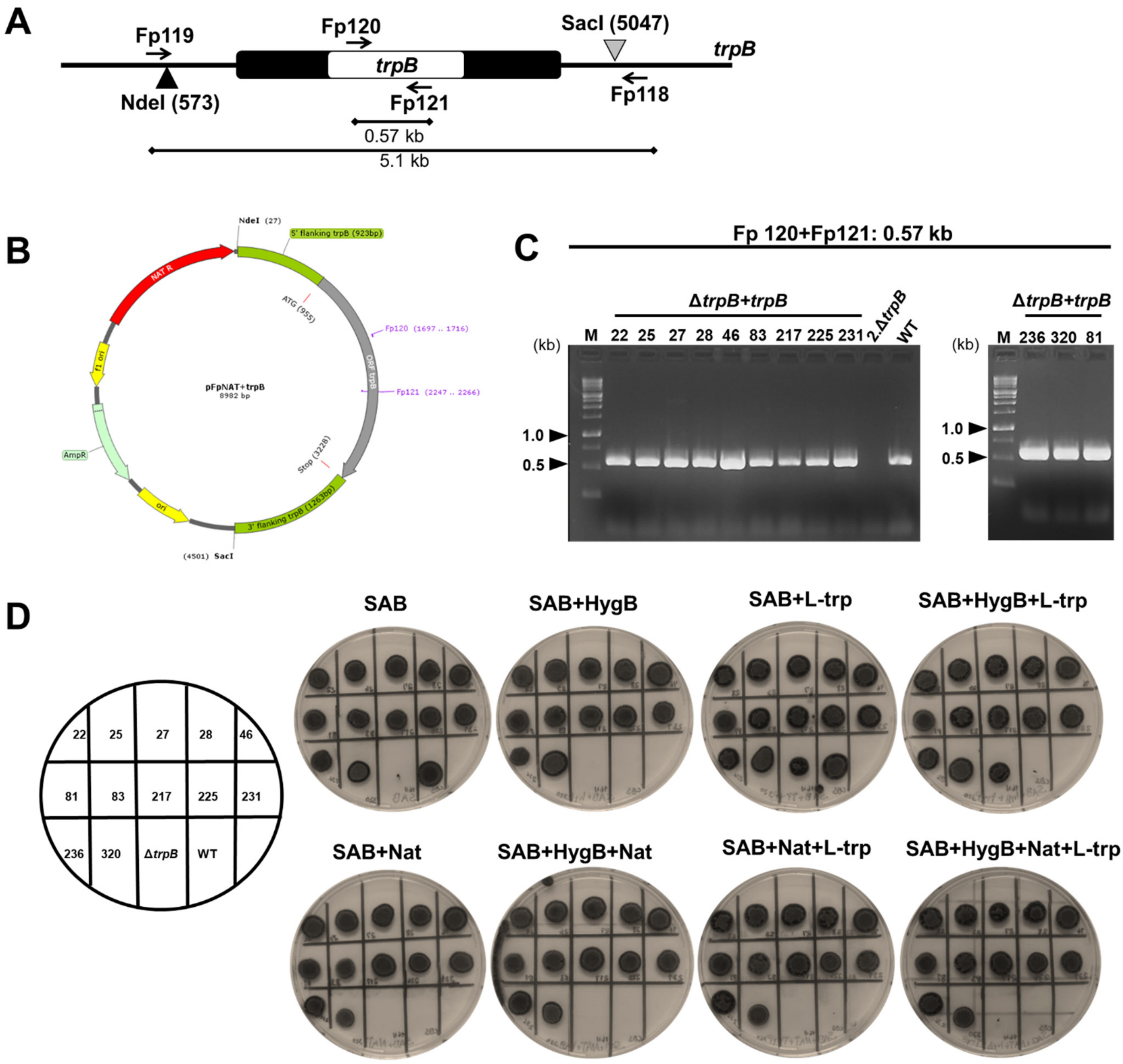

Figure S3. Strategy for obtaining and confirming the complementation of *F. pedrosoi trpB* mutant. (A) Schematic representation of the *trpB* locus indicating the position of the NdeI and SacI restriction sites and the oligonucleotides used in the PCRs. (B) Map of plasmid pFpNAT+*trpB* obtained after cloning the 4.7kb *trpB* into NdeI and SacII restriction sites. (C) Confirmation of 12 reconstituted strains (*trpBΔ* + *trpB)* after PCR amplification using *trpB* internal specific oligonucleotides (Fp120+Fp121). The product sizes are indicated in the picture. (D) Reconstituted strains of *F. pedrosoi* recovered the tryptophan prototrophic phenotype. Twelve transformants were obtained after biolistic transformation of 2.*ΔtrpB* with pFpNAT+trpB were able to grow in the absence of tryptophan after ectopic integration of *trpB* into the genome. The strains were inoculated on Sabouraud agar (SAB), supplemented or not with 500 µg/mL of tryptophan (L-trp), 100 µg/mL Hygromycin B (HygB) and 100 µg/mL Nourseothricin (Nat) and incubated at 28 °C for 7 days. Strains 2. Δ*trpB* and WT (CBS 271.37) were used as controls and are indicated. M: Molecular weight marker, WT: wild type strain, NAT^{R}: Nourseothricin resistance marker, AMP^{R}: Ampicillin resistance marker

TrpB 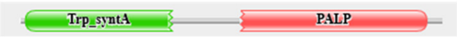

738 aa

Domain	Name	Alignment Position	E-value
Trp_syntA	Tryptophan synthase alpha chain	8 – 258	1.3e-93
PALP	Pyridoxal-phosphate dependent enzyme	389 – 712	2.8e-50

TrpC 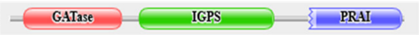

768 aa

Domain	Name	Alignment Position	E-value
GATase	Glutamine amidotransferase class-I	27 – 216	3.6e-46
IGPS	Indole-3-glycerol phosphate synthase	252 – 513	1.0e-95
PRAI	N-(5'phosphoribosyl)anthranilate (PRA) isomerase	598 – 763	7.3e-40

TrpD 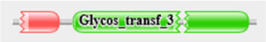

426 aa

Domain	Name	Alignment Position	E-value
Glycos_transf_3	Glycosyl transferase family, a/b domain	110 – 283	1.7e-48

TrpE 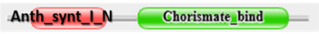

533 aa

Domain	Name	Alignment Position	E-value
Anth_synt_I_N	Anthranilate synthase component I, N terminal	43 – 172	2.5e-25
Chorismate_bind	chorismate binding enzyme	233 – 498	2.3e-89

Figure S4. Conserved signature domains of *F. pedrosoi* putative enzymes required for tryptophan biosynthesis. The domains were searched by pfam tool (https://pfam.xfam.org/search/sequence) in the translated sequences of transcripts identified in the *F. pedrosoi* genome database. The positions of each domain as well as the e-value are shown in the figure.

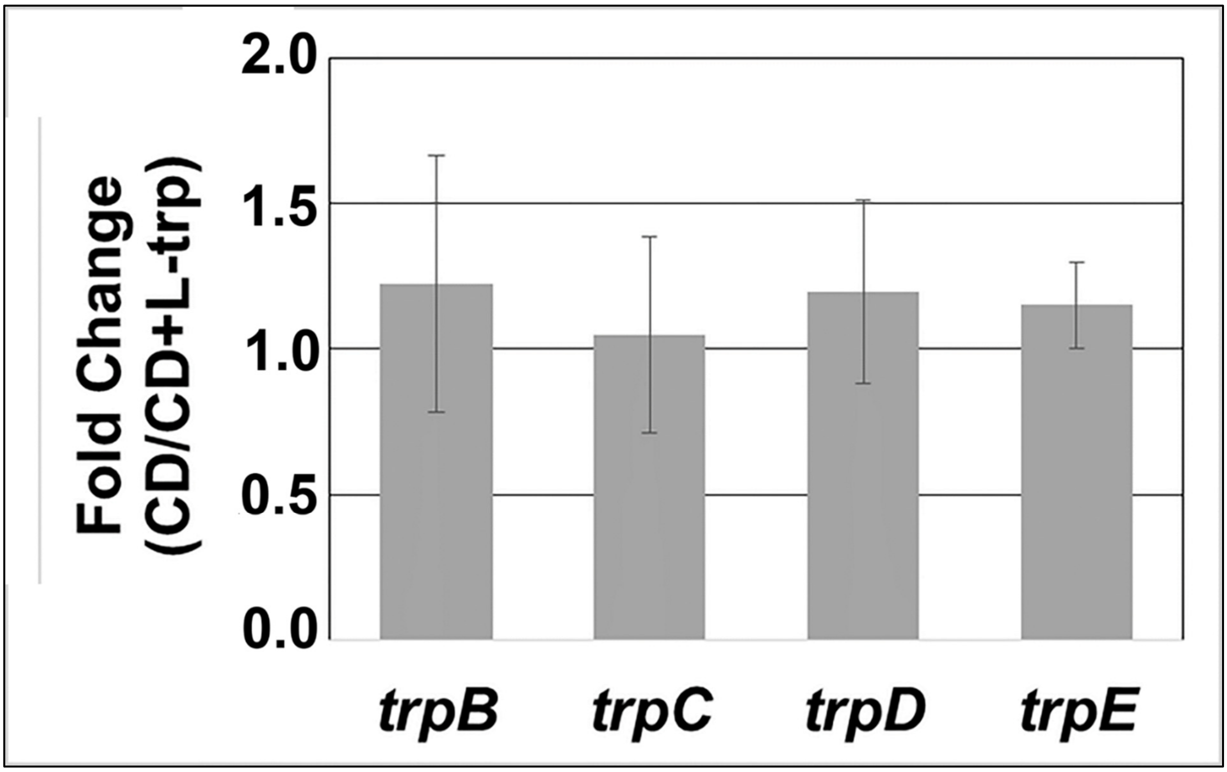

Figure S5. Real time PCR to evaluate the expression of the tryptophan biosynthetic genes: *trpB*, *trpC*, *trpD* and *trpE* of *F. pedrosoi* in response to incubation in minimal medium (Czapeck Dox) for 3h at 28 ºC. Bars represent standard deviation of the mean of three biological replicates. *gapgh* was used as housekeeping gene.

```
KIW78135_TrpB_Fp          -MQAIKETFARCKEEGRTALVGYFTAGYPTAEETPDIMLGLQAGGVDIIELGLPFTDPIA
AAF91181.1_TRPB_An        -MEHIKNTFEKVKREKRAALVAYITAGYPTIEESVDILLGLENGGADIIELGVPFTDPIA
XP_963281.1_Trp-3_Nc      -MEGIKQTFQRCKAQNRAALVTYVTAGFPHPEQTPDILLAMEKGGADVIELGVPFTDPIA
CAA24635.1_trp5_Sc        MSEQLRQTFANAKKENRNALVTFMTAGYPTVKDTVPILKGFQDGGVDIIELGMPFSDPIA
P16578.3_Trp1_Cc          -MEAIKKVFEQKKAQDATAFVAFVTAGYPKKEDTVPVLLALQAGGADIIELGIPFSDPIA
                            : :::.* . * :   *:* :.***:*  :::  :: .:: **.*:****:**:****

KIW78135_TrpB_Fp          DGPTIQKSNTVALKNGITVTSTLQFVRDARKKGLKVPVLFMGYYNPLLRYGEDRMLKDCK
AAF91181.1_TRPB_An        DGPTIQKANTKALANGVTVSTVLEMVQHQRNRGLKAPIMLMGYYNPMLSYGEERMLRDCK
XP_963281.1_Trp-3_Nc      DGPTIQTANTIALQHGVTLQSTLQMVRDARQRGLKAPVMLMGYYNPLLSYGEERLLNDCK
CAA24635.1_trp5_Sc        DGPTIQLSNTVALQNGVTLPQTLEMVSQARNEGVTVPIILMGYYNPILNYGEERFIQDAA
P16578.3_Trp1_Cc          DGPVIQEANTVALKNDIDYPTVLGQIREARQQGLTAPVLLMGYYNPMLAYGEDKAIQDAA
                          ***.** :** ** :.:    .*  : . *:.*:..*:::******:* ***:: :.*.

KIW78135_TrpB_Fp          EAGVNGFIMVDLPPEEAVRFRNNCTKAGLSYVPLIAPATSEKRMKLLCQIADSFIYVVSR
AAF91181.1_TRPB_An        EVGVNGFIMVDLPPEEAVRFRDLCTSTGLSYVPLIAPATSESRMKLLCKIADSFIYVVSR
XP_963281.1_Trp-3_Nc      EAGVNGFIIVDLPPEEAVSFRQLCTRGGLSYVPLIAPATSDARMRVLCQLADSFIYVVSR
CAA24635.1_trp5_Sc        KAGANGFIIVDLPPEEALKVRNYINDNGLSLIPLVAPSTTDERLELLSHIADSFVYVVSR
P16578.3_Trp1_Cc          EAGANGFIMVDLPPEEAIAFRQKCAASNLSYVPLIAPSTTLKRIQFLASIADSFIYVVSK
                          :.*.****:********: .*:     .** :**:**:*:  *:..*. :****:****:

KIW78135_TrpB_Fp          MGVTGATG--TLNTKLPELLERVHQYSGGIPAAVGFGVSTREHFLSVSQIAEG--VVIGS
AAF91181.1_TRPB_An        MGVTGATG--TLSSNIPELLARVHKWSGNVPTALGFGVSTREHFLSVQEISEG--VVIGS
XP_963281.1_Trp-3_Nc      QGVTGASG--TLNANLPELLARVKKYSGNKPAAVGFGVSTHDHFTQVGAIADG--VVVGS
CAA24635.1_trp5_Sc        MGTTGVQS--SVASDLDELISRVRKYTKDTPLAVGFGVSTREHFQSVGSVADG--VVIGS
P16578.3_Trp1_Cc          MGTTGSSANVAVNEELPTILSRIREYT-HVPLAVEFGVATRDQFNYVADAGADGVVVIGS
                           *.**  .  ::  .:  :: *:::::   * *: ***:*:::*  *   . .  **:**

KIW78135_TrpB_Fp          QIVTVLGNAKQGERAKAVQEYCSQVTGRKLGDFNTTDGLTREVGIVETMKSAKEPNAAAN
AAF91181.1_TRPB_An        QIITVLGEAPAGQAAQKAEEYLSSVTGRKLERD-TSGALTREVNVLEPVLKIETP-----
XP_963281.1_Trp-3_Nc      MIITTIQKAAKGEEVKAVQEYCSYLCGRNFEQS---------------------------
CAA24635.1_trp5_Sc        KIVTLCGDAPEGKRYDVAKEYVQGILN---------------------------------
P16578.3_Trp1_Cc          RIVNAIKAAGEGQVPQFVENYCREVSGKGEPSR---------------------------
                           *:.    *  *:  . .::*   : .

KIW78135_TrpB_Fp          GLNGHQVQVDKVITDADVPSEPGLADQLDALNSATNGTAPNPDAIPARFGQFGGQYVPES
AAF91181.1_TRPB_An        ----PMSQPTDVITEKDTPSGPGLGDQLEALNGGGS-----SSATPARFGEFGGQYVPES
XP_963281.1_Trp-3_Nc      ---AHELNMGEALEAAKEPVGTATVDGVITEADIDAQLAALHGTIPKRFGEFGGQYVPEA
CAA24635.1_trp5_Sc        ------GAKHKVLSKDEFFAFQKESLKSANVKKEILDEFDENHKHPIRFGDFGGQYVPEA
P16578.3_Trp1_Cc          -----------------VRSPGAAQRTPSQLTPNAETAKGVENILPARFGQFGGQYVPES
                                                                       * ***:********:

KIW78135_TrpB_Fp          LMDCLAELEEGFQKAKNDPEFWKEYKSYYPYMGRPSSLHLADRLTERVGGAQIYLKREDL
AAF91181.1_TRPB_An        LMDCLAELERGFDSALKDPSFWEEYRSYYPYMGRPSSLHLATRLTEHVGGANIWLKREDL
XP_963281.1_Trp-3_Nc      LMDCLSELEEGFNKIKDDPAFWEEYRSYYPWMGRPGQLHKAERLTEYAGGANIWLKREDL
CAA24635.1_trp5_Sc        LHACLRELEKGFDEAVADPTFWEDFKSLYSYIGRPSSLHKAERLTEHCQGAQIWLKREDL
P16578.3_Trp1_Cc          LVDALAELEEAHKSAIEDPAFWEEVRSLYTYSNRPSNLYLAENLTKEAGGANIWLKREDL
                          *  .* ***.....   ** **:: :* *.: .**..*: * .**:   **:*:******

KIW78135_TrpB_Fp          NHTGSHKINNALGQILLARRLGKTRIIAETGAGQHGVATATVCAKFGMACVVYMGAEDVR
AAF91181.1_TRPB_An        NHTGSHKINNALGQILLARRLGKTRIIAETGAGQHGVATATVCAKFGMKCVVYMGAEDVR
XP_963281.1_Trp-3_Nc      NHTGSHKINNALGQLLLARRLGKKKIIAETGAGQHGVATATVCAKFGMECTVFMGAEDVR
CAA24635.1_trp5_Sc        NHTGSHKINNALAQVLLAKRLGKKNVIAETGAGQHGVATATACAKFGLTCTVFMGAEDVR
P16578.3_Trp1_Cc          NHTGSHKINNALGQILLAKRIGKTRIIAETGAGQHGVATATVCAKFGLECVIYMGAEDVR
                          ************.*:***:*:**..:***************.*****: *.::*******

KIW78135_TrpB_Fp          RQALNVFRMKLLG--AKVVAVEAGSRTLRDAVNEALRAWVVDLDTTHYIIGSAIGPHPFP
AAF91181.1_TRPB_An        RQALNVFRMKLLG--ASVVAVDAGSRTLRDAVNEALRAWVVDLDTTHYIIGSAIGPHPFP
XP_963281.1_Trp-3_Nc      RQALNVFRMKLLG--AKVVAVEAGSRTLRDAVNEALRYWVVNLADTHYIIGSAIGPHPFP
CAA24635.1_trp5_Sc        RQALNVFRMRILG--AKVIAVTNGTKTLRDATSEAFRFWVTNLKTTYYVVGSAIGPHPYP
P16578.3_Trp1_Cc          RQALKLFRIEMLGGKAWVIPVHSGSCTLKDAVNEAMRDWVTNLSTTHYLVGSAIGPHPFP
                          ****::**:.:**  * *:.*  *: **:**..**:* **.:*  *:*::********:*

KIW78135_TrpB_Fp          TIVRTFQSVIGQETKEQMKELT-GRLPDAVIACVGGGSNAVGMFFPFSSDPSVKLIGVEA
AAF91181.1_TRPB_An        TIVRTYQSVIGEETKQQLQEQI-GKLPDAVVACVGGGSNAVGSFYPFSKDTSVKLLGVEA
XP_963281.1_Trp-3_Nc      TIVRTFQSVIGNETKQQMLEKR-GKLPDAVVACVGGGSNAVGMFYPFSNDPSVKLLGVEA
CAA24635.1_trp5_Sc        TLVRTFQSVIGKETKEQFAAMNNGKLPDAVVACVGGGSNSTGMFSPFEHDTSVKLLGVEA
P16578.3_Trp1_Cc          TIVRDFQKVIGEEIKAQLKEVR-GKLPDVVVACVGGGSNAIGTFYDFIPDKSVRLVGVEA
                          *:** :*.***:* * *:     *:***.*:********: * *  *  * **:*:****

KIW78135_TrpB_Fp          AGEGVDTGRHSATLSGGSIGVLHGVRTYVLQNEHGQISDTHSISAGLDYPGVGPELSNWK
AAF91181.1_TRPB_An        GGDGIDTNRHSATLSGGSKGVLHGVRTYILQDEHGQVSETHSISAGLDYPGVGPELSNWK
XP_963281.1_Trp-3_Nc      GGDGVDTPRHSATLTAGSKGVLHGVRTYILQNQYGQIEDTHSISAGLDYPGVGPELSNWK
CAA24635.1_trp5_Sc        GGDGVDTKFHSATLTAGRPGVFHGVKTYVLQDSDGQVHDTHSVSAGLDYPGVGPELAYWK
P16578.3_Trp1_Cc          GGEGIDGHKHSATLSMGQPGVLHGVRTYILQDKAGQIIETHSISAGLDYPGVGPEHAWLK
                          .*:*:*   *****: *  **:***:**:**:. **: :***:************ :  *

KIW78135_TrpB_Fp          DISRARFISATDSEALIGFRTLSETEGIIPALESSHAIFGAMQVAKQMNKDQTLVICLSG
AAF91181.1_TRPB_An        DSDRAHFIAATDAQALSGFRTMAQTEGIIPALESSHAIWGAQELAKTMKGKGDIVLTVSG
```

```
XP_963281.1_Trp-3_Nc      DTERAKFVAATDAQAFEGFRLMSQLEGIIPALESSHGIWGALELAKTMKPDEDVVICLSG
CAA24635.1_trp5_Sc        STGRAQFIAATDAQALLGFKLLSQLEGIIPALESSHAVYGACELAKTMKPDQHLVINISG
P16578.3_Trp1_Cc          DSGRADYVVCTDEDALRGFRMLTQKEGIIPALESSHAIWEGVKIAKSLPKDKDIVICLSG
                          .  ** :: .** :*: **: ::: ***********.:: . ::** :  .  :*: :**

KIW78135_TrpB_Fp          RGDKDVQSVADELPRLGPGIGWDLRF------
AAF91181.1_TRPB_An        RGDKDVESVAESLPKLGPQIGWDLRF------
XP_963281.1_Trp-3_Nc      RGDKDVQSVADELPIIGPKIGWDLRF------
CAA24635.1_trp5_Sc        RGDKDVQSVAEVLPKLGPKIGWDLRFEEDPSA
P16578.3_Trp1_Cc          RGDKDVEQISELLPKWADKLDWHVSSNAIPSK
                          ******:.:::  **  .   :.*.:
```

Figure S6. Multiple sequence alignment of tryptophan synthases protein sequences. The analyses of the sequences from *F. pedrosoi* (KIW78135), *Aspergillus nidulans* (AAF91181.1), *Neurospora crassa* (XP_963281.1), *Saccharomyces cerevisiae* (CAA24635.1) and *Coprinopsis cinerea* (P16578.3) was performed on CLUSTALW server (https://www.genome.jp/tools-bin/clustalw). The connecting region delimited by Tyrosine (Y) and Proline (P) between α and β catalytic domains are in bold. The access numbers to the NCBI deposited sequence are in parentheses.

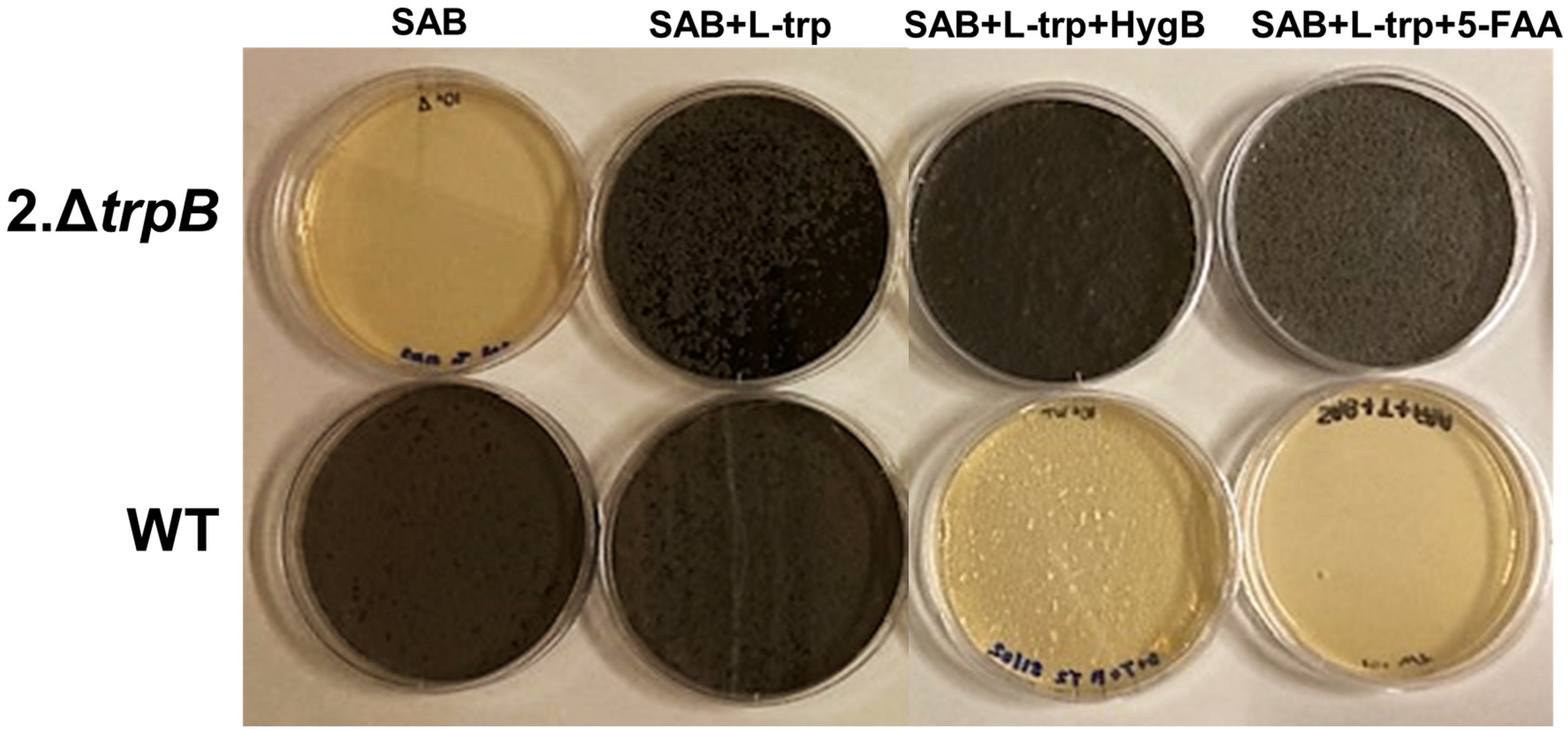

Figure S7. Evaluation of the use of 5-FAA at the concentration of 0.5 mg/mL as a selection agent for *trp*⁻ mutants, and for counter-selection of strains that present active tryptophan synthesis pathway at high cell density (5×10^5) of WT and Δ*trpB*. The plates SAB agar, SAB agar +L-trp, SAB agar+L-trp+HygB and SAB agar+L-trp+5-FAA were incubated at 25 ^{o}C and inspected every three days until the 22nd day of growth. SAB agar plates were supplemented with 500 µg/mL of L-trp and/or 100 µg/mL of HygB.
